# Triazole RGD antagonist reverts TGFβ1-induced endothelial-to-mesenchymal transition in endothelial precursor cells

**DOI:** 10.1007/s11010-016-2847-2

**Published:** 2016-10-19

**Authors:** Francesca Bianchini, Silvia Peppicelli, Pierangelo Fabbrizzi, Alessio Biagioni, Benedetta Mazzanti, Gloria Menchi, Lido Calorini, Alberto Pupi, Andrea Trabocchi

**Affiliations:** 1 Department of Clinical and Experimental Biomedical Science “Mario Serio”, University of Florence, Florence, Italy; 2 Department of Chemistry “Ugo Schiff”, University of Florence, Florence, Italy; 3 Cord Blood Bank, Careggi University Hospital, Florence, Italy; 4 Interdepartmental Center for Preclinical Development of Molecular Imaging (CISPIM), University of Florence, Florence, Italy

**Keywords:** αvβ3 integrin, Endothelial Colony-Forming Cells (ECPCs), Endothelial-to-Mesenchymal Transition (EMT), Fibrosis, Transforming Growth Factorβ1 (TGFβ1)

## Abstract

Fibrosis is the dramatic consequence of a dysregulated reparative process in which activated fibroblasts (myofibroblasts) and Transforming Growth Factor β1 (TGFβ1) play a central role. When exposed to TGFβ1, fibroblast and epithelial cells differentiate in myofibroblasts; in addition, endothelial cells may undergo endothelial-to-mesenchymal transition (EndoMT) and actively participate to the progression of fibrosis. Recently, the role of αv integrins, which recognize the Arg-Gly-Asp (RGD) tripeptide, in the release and signal transduction activation of TGFβ1 became evident. In this study, we present a class of triazole-derived RGD antagonists that interact with αvβ3 integrin. Above different compounds, the RGD-2 specifically interferes with integrin-dependent TGFβ1 EndoMT in Endothelial Colony-Forming Cells (ECPCs) derived from circulating Endothelial Precursor Cells (ECPCs). The RGD-2 decreases the amount of membrane-associated TGFβ1, and reduces both ALK5/TGFβ1 type I receptor expression and Smad2 phosphorylation in ECPCs. We found that RGD-2 antagonist reverts EndoMT, reducing α-smooth muscle actin (α-SMA) and vimentin expression in differentiated ECPCs. Our results outline the critical role of integrin in fibrosis progression and account for the opportunity of using integrins as target for anti-fibrotic therapeutic treatment.

## Introduction

Fibrotic disease encloses a wide array of different pathologies both systemic such as systemic sclerosis (SSc) and sclerodermatous graft versus host disease (Scl GVHD), and organ-specific pathologies as idiopathic pulmonary fibrosis (IPF), liver cirrhosis, and progressive kidney disease. Although the etiology of these fibrotic disorders remains unexplained and may vary from disease to disease, the common pathogenetic signs are the deposition of extracellular matrix (ECM) synthesized by activated myofibroblasts and the persistence of inflammation [1, 2]. High levels of cytokines, growth factors, and proteolytic enzymes produced by activated macrophages and lymphocytes restrain the resolution process and fibrosis expands gradually. Finally, matrix deposition rearranges the tissue architecture, causing organ failure and patient death. Thus, fibrotic diseases in their complex are life-tethering diseases recognized to represent an important health issue [3].

Transforming growth factorβ1 (TGFβ1) has been identified as the most important mediator in many types of tissue fibrosis [4]. TGFβ1 induces fibroblasts differentiation into myofibroblasts providing collagen and ECM protein deposition [5]. Experimental studies have provided evidences that myofibroblasts not only originate from resident fibroblasts, but also derive from the transdifferentiation of epithelial cells, endothelial cells, and bone marrow-derived cells [6, 7]. Endothelial cells and bone marrow-derived endothelial cells, when exposed to TGFβ1, undergo endothelial–mesenchymal transition (EndoMT). As a consequence, endothelial cells lose their biological characteristic of the endothelial phenotype, acquire typical mesenchymal features, and concurrently express typical markers of myofibroblastic differentiation: α-smooth muscle actin (α-SMA), vimentin, and collagen production [8].

TGFβ1 is produced as inactive pro-peptide by different types of inflammatory cells. The mature cytokine is exposed in the extracellular space non-covalently associated to a latency-associated peptide (LAP), and the LAP–TGFβ1 pro-peptide forms a homodimer complex. This complex, which prevents the interaction of the mature cytokine with its receptors, is stored in the extracellular space, closely associated to the cell membrane and bounded to specific latent TGFβ1 binding proteins (LTBP). LTBP fixes the system in association to fibrillin-1 and fibronectin. The release of the non-covalently bounded mature TGFβ1 from the LAP complex occurs by the action of proteases (matrix metalloproteases MMP, thrombin, and plasmin) or by the effect of environmental condition changes (pH, pO2, or temperature) [9, 10]. Moreover, in the recent years, the role of αv integrins in TGFβ1 release from the LAP complex during fibrotic disease progression became evident [11, 12]. It has been demonstrated that αv integrins recognize the tripeptide Arg-Gly-Asp (RGD) sequence of the LAP, and the integrin binding induces a stretch of the complex and a mechanical release of the active TGFβ1 [13]. In addition, it is known that αv integrins might function as docking site for MMPs contributing to the protease-dependent release of TGFβ1. Thus, the maintenance of high levels of TGFβ1 exacerbates tissue damages and outlines the important role of integrins in the instauration and progression of fibrosis [14, 15].

In this study, we present a novel class of αvβ3 RGD antagonists that interfere with integrin-dependent release of TGFβ1 from LTBP complex. Interestingly, we found that the RGD-2-integrin antagonist counteracts the TGFβ1-induced myofibroblast differentiation of endothelial precursor cells (ECPCs) by interfering, in these cells, with the autocrine loor of TGFβ1 activation.

These findings may suggest an innovation in anti-fibrotic treatment. The common anti-fibrotic treatments are based, so far, on the use of corticosteroids and immunosuppressant drugs. These drugs may retard, but do not arrest, the disease progression. Thus, the cooperation between anti-fibrotic available drugs and novel RGD antagonists might contribute to the inhibition of the vicious cycle, led by TGFβ1, underlying the disease progression.

## Results

### RGD triazole-derived αvβ3 antagonists

RGD triazole-derived antagonists of the αvβ3 integrin receptor were synthesized in our laboratory as previously described [16]. Solid-phase assay was used to test the ability of RGD ligands to compete with ^125^I-echistatin for the binding to αvβ3 integrin receptor. We found that RGD-1 compound showed an IC50 value of 2.1 ± 1.3 μM, while compound RGD-2 showed an IC50 of 37 ± 11 nM. In contrast, the IC50 value of RGD-3 compound was found to be >10 μM, addressing for a less active antagonistic efficacy toward αvβ3 receptor (Table 1).

**Table 1:** Inhibition of ^125^I-echistatin-specific binding to purified human integrin proteins αvβ3 by triazole-containing RGD peptidomimetics

Triazole-derived RGD antagonist	Structure	Specific binding versus αvβ3, IC50 (μM)
RGD-1^16^		2.1 ± 1.3
RGD-2^16^		0.037 ± 0.011
RGD-3^18^		>10

### Effect of the triazole RGD antagonists on ECPCs adhesion

**Fig. 1:**
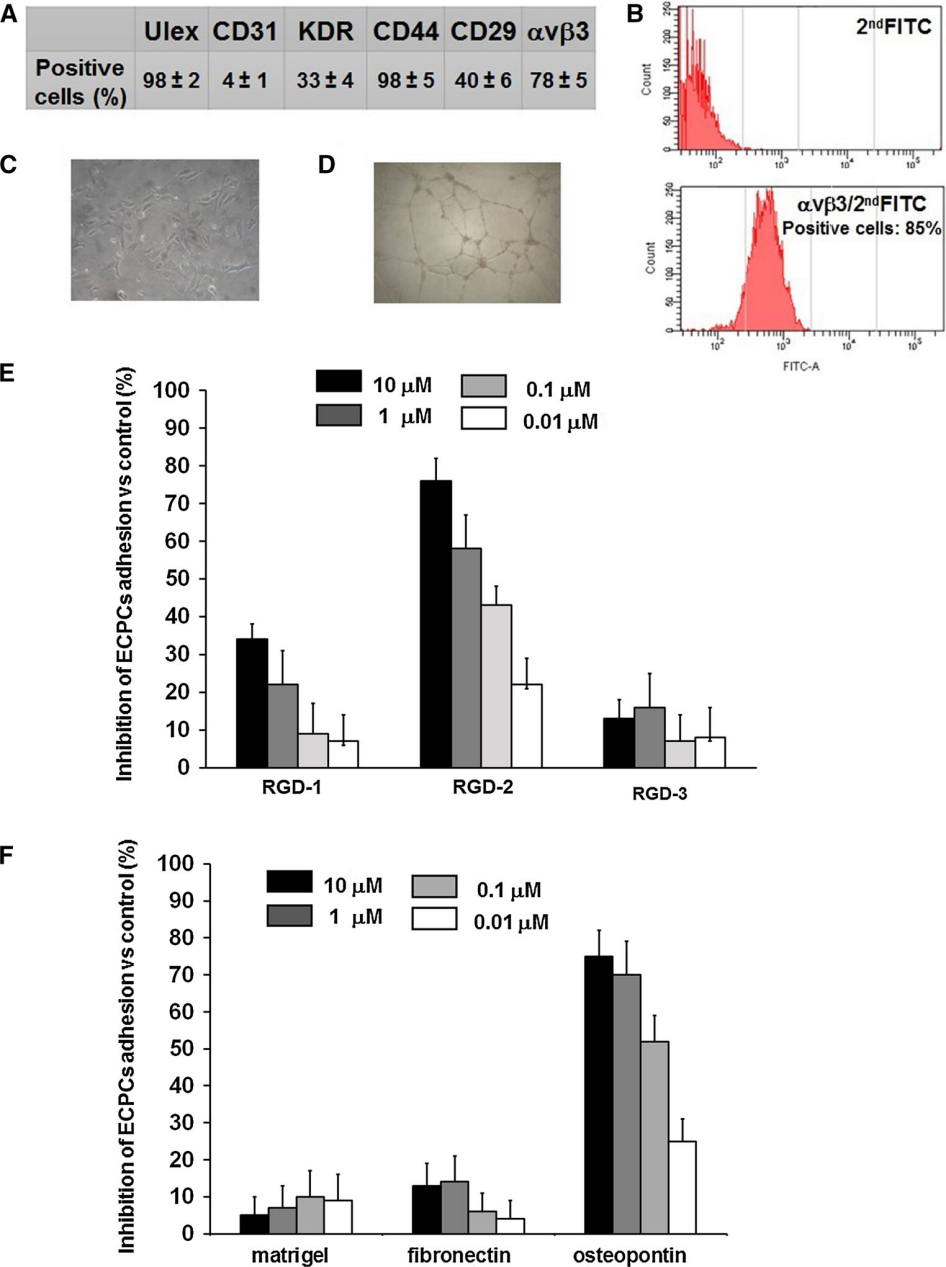
RGD triazole-derived antagonists on ECPCs adhesion to RGD containing substrata. **a** Flow cytometric analysis of surface antigens expression in freshly isolated ECPCs (percentage of positive cells). Biological properties of ECPCs, **b** flow cytometric analysis of αvβ3 integrin expression of cultured cells, **c** contrast microscopy images of ECPCs growing cultures, and **d** contrast microscopy images of ECPCs tube formation. **e** Inhibition of adhesion of ECPCs to VN in the presence of different concentration of different RGD triazole antagonists (RGD-1, RGD-2, and RGD-2 triazole compounds), and **f** inhibition of adhesion of ECPCs to Matrigel, FN, and OPN in the presence of different concentrations of RGD-2 antagonist. Representative results from three different experiments. Values represent the mean ± SD

ECPCs, isolated from human umbilical cord blood (UCB), were characterized using flow cytometry assay by surface expression of endothelial cell-specific antigens: Ulex europaeus I agglutinin (Ulex), Platelet Endothelial Cell Adhesion Molecule-1 (PECAM-1/CD31), Vascular Endothelial Growth Factor Receptor 2 (VEGFR-2/KDR), Phagocytic Glycoprotein-1 (CD44), integrin β1-chain (CD29), and integrin heterodimers αvβ3 (Fig. 1a). ECPCs were monitored throughout the experimental procedures for αvβ3 integrin expression (Fig. 1b), the maintenance of endothelial phenotype (Fig. 1c), and the in vitro tube formation ability (Fig. 1d). Sub-confluent cultures of ECPCs between the 3rd and 6th passage were exposed to different doses (10, 1, 0.1, and 0.01 μM) of three different triazole RGD antagonists and allowed to adhere to vitronectin (VN) for 1 h. At the end of the incubation, non-adherent cells were removed by a gentle wash with PBS. We found a 40 % of inhibition of ECPCs adhesion to VN for RGD-1 compound at 10 μM, whereas lower concentrations of this compound showed a weak inhibition of cells adhesion. The RGD-2 compound clearly inhibited ECPCs adhesion to VN in a dose-dependent manner, ranging from 80 % of inhibition at 10 μM to 20 % at 10 nM. Finally, RGD-3 compound did not significantly inhibit ECPCs adhesion to VN (Fig. 1e). To further evaluate RGD-2 inhibitory activity toward different RGD containing substrata, ECPCs cells were exposed to RGD-2 compound at different doses before adhesion to osteopontin (OPN), Fibronectin (FN), and Matrigel. We found that RGD-2 antagonist showed a dose-dependent inhibition of ECPCs adhesion to OPN from 75 % at 10 μM to 25 % at 10 nM. In contrast to this, inhibition of adhesion to FN and Matrigel was very weak. Matrigel was used as negative control since it is composed by laminin, collagen IV, nidogen/enactin, and proteoglycan, which are ligands for other non-RGD families of integrin receptors (laminin-type and collagen-type) (Fig. 1f).

### Effect of RGD-2 triazole compound on αvβ3 expression in TGFβ1-stimulated ECPCs

**Fig. 2:**
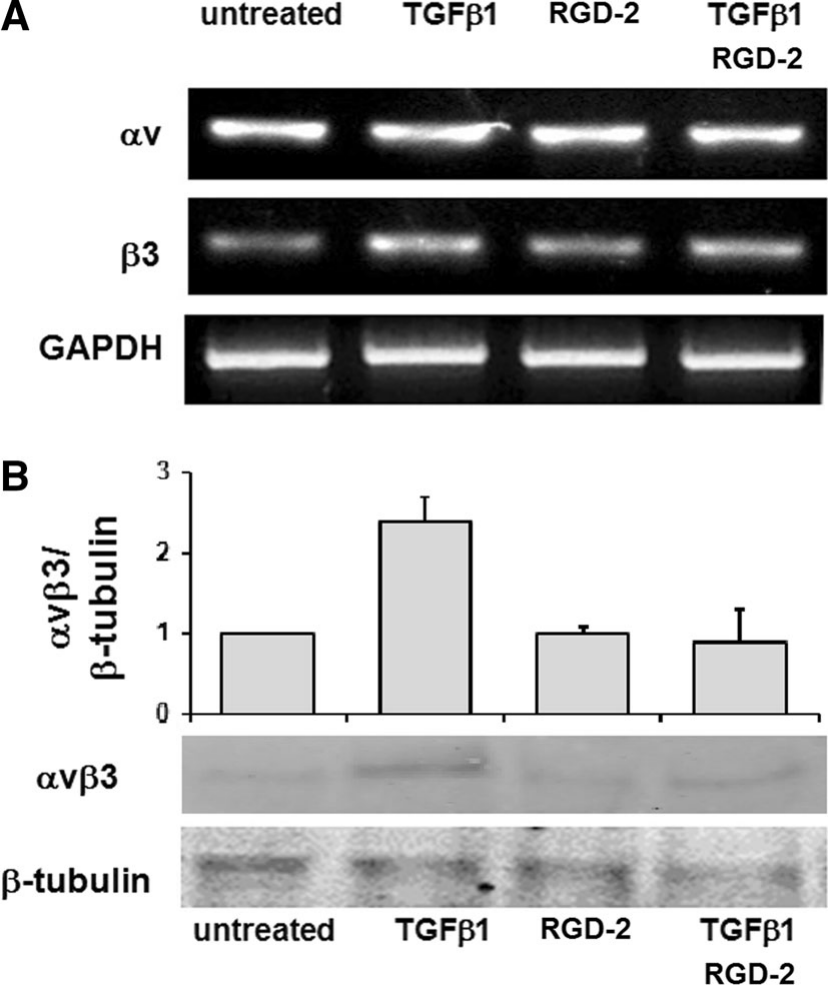
Effect of RGD-2 on αvβ3 expression in ECPCs. Integrin αvβ3 expression in ECPCs after 24 h treatment with exogenous TGFβ1 (1 ng/ml) and/or 1 μM RGD-2 antagonist: **a** mRNA for αv, β3 subunits, and GAPDH, and **b** αvβ3 protein expression and densitometric analysis

ECPCs were exposed to RGD-2 compound at 1 μM and/or TGFβ1 at 1 nM/ml for 24 h. Here we found an overexpression of β3 subunit mRNA in TGFβ1-treated ECPCs, while the co-treatment with the RGD-2 antagonist did not show any β3 subunit increase (Fig. 2a). Along with this, we found that protein expression of αvβ3 receptor was increased, in TGFβ1 treated ECPCs, while treatment with RGD-2 antagonist abrogates TGFβ1, inducing αvβ3 expression in ECPCs (Fig. 2b).

### Effect of RGD-2 antagonist on TGFβ1 signal transduction pathway and TGFβ1 expression on ECPCs cell membrane

**Fig. 3:**
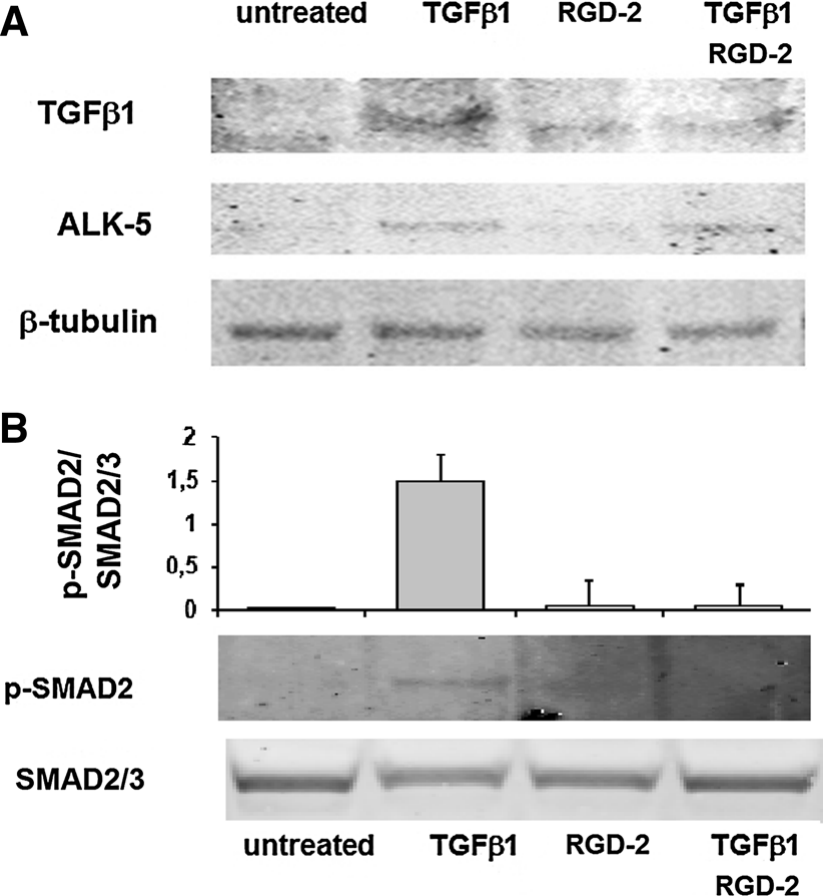
Effect of RGD-2 antagonist on TGFβ1 signal transduction pathway and TGFβ1 expression on ECPCs cell membrane. **a** TGFβ1 and ALK-5 were evaluated in ECPCs exposed for 24 h to exogenous TGFβ1 and/or RGD-2 triazole and for additional 24 h to fresh standard medium. **b** Protein expression and densitometric analysis of PhosphoSMAD2 (pSMAD2) evaluated in ECPCs exposed to exogenous TGFβ1 and/or RGD-2 for 1 h. All experiments were conducted at least three times. Values represent the mean ± SD. *P < 0.05

In order to evaluate the signal transduction activation of TGFβ1 pathway in ECPCs cells, we investigated ALK-5 expression and SMAD phosphorylation after TGFβ1 and/or RGD-2 treatment. We observed that 24 h treatment with TGFβ1 induces the increase of ALK-5 expression also in the presence of the RGD antagonist (Fig. 3a). Moreover, when we evaluated SMAD2 phosphorylation, we found an increase in Phospho-SMAD2 in TGFβ1-treated cells, while in RGD-2/TGFβ1-co-treated cells, SMAD2 phosphorylation was similar to untreated cells (Fig. 3b). We, also, evaluated the effect of RGD-2 antagonist on TGFβ1 activation in ECPCs. Cells were exposed for 24 h to exogenous TGFβ1 (1 ng/ml). After the removal of the medium, cells were allowed to grow for the next 24 h in a standard medium. At the end of the second incubation, ECPCs were lysed directly in tissue culture plates and processed for western blotting analysis. We found high levels of membrane-associated TGFβ1, in TGFβ1-treated cells, compared to untreated cells. While the treatment with RGD-2 antagonist (1 μM) alone did not modify TGFβ1 levels, the co-treatment with TGFβ1 and RGD-2 antagonist reduced significantly the expression of endogenous TGFβ1 to the levels found in untreated cells (Fig. 3a).

### RGD-2 antagonist reverts TGFβ1-induced EndoMT in ECPCs

**Fig. 4:**
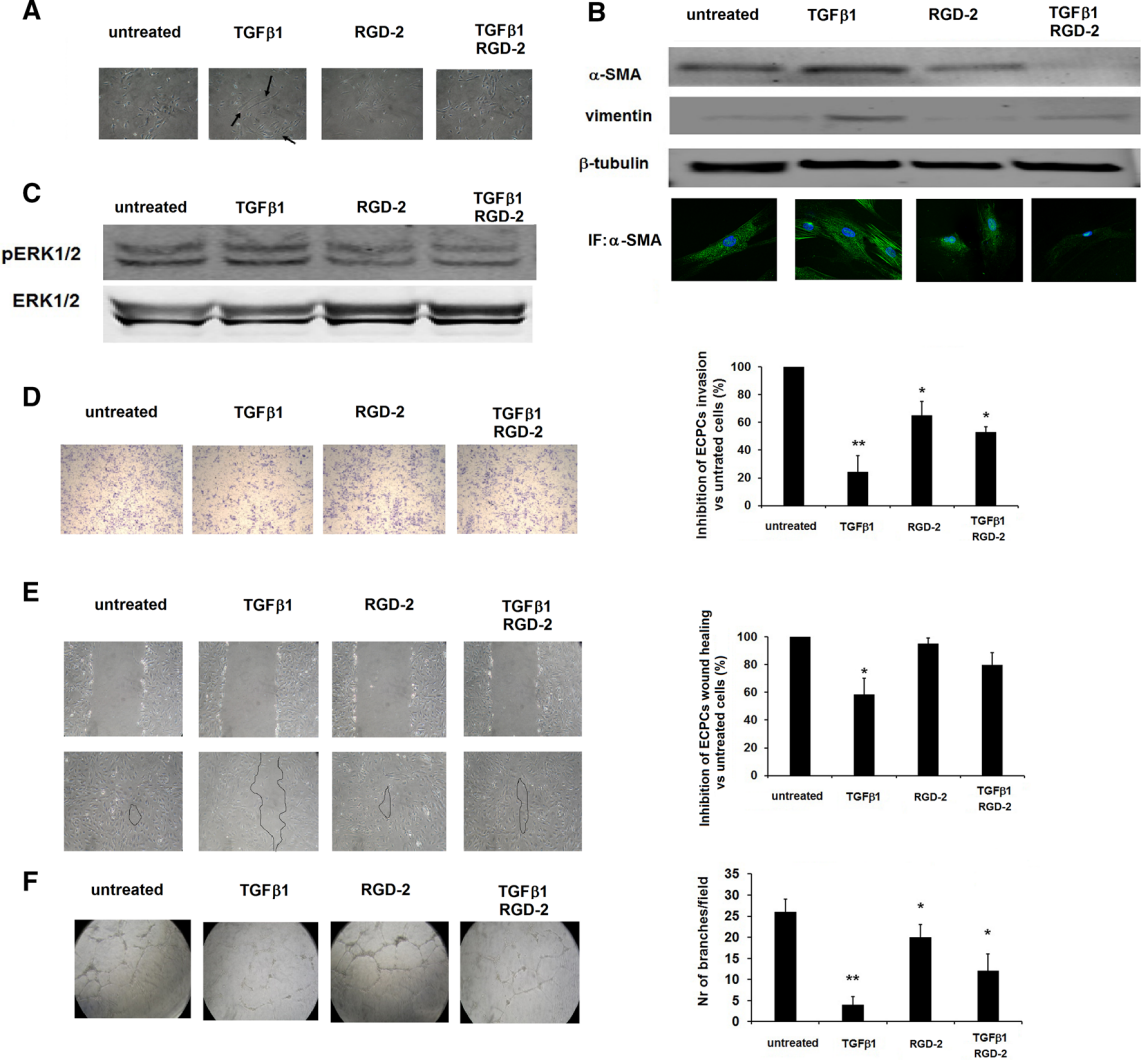
RGD-2 antagonist reverts TGFβ1-induced EndoMT in ECPCs. **a** Contrast microscopy representative images of ECPCs after 24 h treatment with TGFβ1 and/or RGD-2 compound, morphological changes. **b** Expression of EndoMT markers of mesenchymal differentiation; ECPCs were exposed to exogenous TGFβ1 (1 ng/ml) and/or 1 μM RGD-2 antagonist for 24 h, and α-SMA and vimentin expression were evaluated. *Upper panel*: western blotting for α-SMA and vimentin; *lower panel*: representative immunofluorescence images for α-SMA. **c** TGFβ1-induced phosphoERK1/2 activation, ECPCs were exposed to different treatments for 24 h and lysed. **d** Invasiveness through Matrigel of ECPCs after 24 h treatment with exogenous TGFβ1 (1 ng/ml) and/or 1 μM RGD-2 antagonist; for quantification, migrated cells were counted in six randomly chosen fields for each filter. **e** Wound healing assay of ECPCs after 24 h treatment with exogenous TGFβ1 (1 ng/ml) and/or 1 μM RGD-2 antagonist; the degree of healing was quantified by measuring the distance between opposing edges of the wound. Four wound/treatment and three measurements/wound were taken. **f** In vitro tube formation of ECPCs after 12 h treatment with exogenous TGFβ1 (1 ng/ml) and/or 1 μM RGD-2 antagonist; for quantification, the number of branches per field was evaluated at 40 magnifications, in four different fields. Data were obtained from three independent experiments. Percentage of inhibition was expressed compared to untreated cells. Values represent the mean ± SD. *P < 0.05, **P < 0.01

We found that TGFβ1 significantly increased the expression of α-SMA and vimentin in ECPCs together with a significant switch to an elongated morphology. Upon the morphological observation of ECPC-treated cultures, we found that TGFβ1-treated cells present a fibroblast-like morphology, as expected, but this change was absent when the cells were treated with the RGD antagonist and TGFβ1, and the culture morphology was slightly similar to that of untreated cells (Fig. 4a). RGD-2 antagonist-treated cells express α-SMA and vimentin at levels similar to the untreated cells. Thus, the treatment with the RGD-2 antagonist in association with TGFβ1 significantly reduced the expression of mesenchymal markers of EndoMT, as demonstrated in western blotting analysis and in immunofluorescence (Fig. 4b). Next, we evaluated MAPK pathway activation after exposure to TGFβ1 and to the co-treatment TGFβ1/RGD-2. We found that ERK1/2 phosphorylation increases in ECPCs after treatment with exogenous TGFβ1, and that the co-treatment with TGFβ1/RGD-2 inhibited ERK1/2 phosphorylation (Fig. 4c). To explore the effect of RGD antagonist on ECPCs biological behavior during TGFβ1-induced EndoMT, we evaluated the invasive activity, wound healing ability, and in vitro angiogenesis of cells exposed to exogenous TGFβ1 and RGD-2 antagonist. We found a significant reduction in ECPCs invasion through Matrigel after treatment with exogenous TGFβ1. Significant but less intense reduction in ECPCs invasion was found after treatment with RGD-2 antagonist alone, while the treatment with RGD antagonist together with TGFβ1 partially restored the originally migratory phenotype of the cells (Fig. 4d). Moreover, it is widely recognized that ECPCs exert a fundamental role the healing process, restoring the physiological function of vascular network. We found that, accordingly with previous finding, ECPCs cells exposed to TGFβ1 lose their wound healing potency, while ECPCs cells treated with TGFβ1 and RGD-2 antagonist almost completely recover their ability to heal the wound. Finally, it is known that ECPCs are characterized by their high proliferation rate and by their ability to give rise to capillary network in the in vitro angiogenic assay; thus, ECPCs were seeded on Matrigel in the presence of TGFβ1 and/or the RGD-2 antagonist. After 24 h incubation, the number of branches per field was counted. We observed a significant decrease in the number of branches of ECPCs treated with TGFβ1 as compared to untreated cells. The co-treatment with the RGD antagonist partially restored the ECPCs ability to form a capillary network (Fig. 4f).

## Discussion

In the present study, we investigated the role of different triazole-derived RGD antagonists in the TGFβ1 loop of ECPCs activation during endothelial–mesenchymal transition (EndoMT). Above the different triazole-derived compounds, we found that the stereochemistry of the tyrosine/phenyl moiety was crucial for the antagonistic efficiency of the compounds. The guanidine substitution, in the arginine mimetic portion of the molecule with the aminopyridine moiety, increased the binding affinity toward αvβ3 integrin. Integrin αvβ3 specifically recognizes the Arg-Gly-Asp (RGD) tripeptide present in the sequence of different extracellular matrix proteins. Although ECPCs express the integrin receptors αvβ3 and α5β1 which recognize RGD extracellular matrix proteins as vitronectin (VN), fibronectin (FN), and osteopontin (OPN), in our conditions, ECPCs adhesion to FN was not inhibited by the RGD antagonist; this result might be explained outlining two observations. The first is that αvβ3 and α5β1, even though recognize the RGD tripeptide sequence, display specific binding affinity for a given ligand depending also on few essential amino acid residues surrounding the binding site pocket of the integrin receptor itself; thus, VN and OPN exhibit higher binding affinity for αvβ3 integrin, while FN exhibits high binding affinity to α5β1 integrin [17]. The second observation is that different RGD antagonist might display a different affinity to the same integrin receptor depending on those interactions with receptor amino acid residues close to the binding pocket [18].

Many clinical and experimental observations confirm the key role of TGFβ during the instauration and the persistence of the fibrotic disease, mostly through the autocrine loop of TGFβ activation [19]. The TGFβ belongs to a ligand superfamily comprising the three forms of TGFβs (TGFβ1, TGFβ2, and TGFβ3), Activins, BMPs (Bone Morphogenetic Proteins), and GDFs (Growth and Differentiation Factors). In general, TGFβ superfamily ligands bind to a complex of TGFβ type I and TGFβ type II serine/threonine kinase receptors. In the absence of stimuli, the homodimers of type I, also known as Activin receptor‐Like Kinase (ALK), and of type II receptors are expressed on the cell surface in a separate form [20].

Upon recognition of the TGFβ1 by the type II receptor, whose function is to present the ligand, the activation of the downstream signal requires the association of the type I/type II receptors in a heterotetrameric complex and the transphosphorylation of the type I receptor/ALK by the serine/threonine kinase activity of type II receptor. Then, the type I receptor transfers the signal into the nucleus by the phosphorylation of the SMAD proteins. The combination of different type II/type I tetrameric receptors determines the activation of different downstream signaling pathways, in response to the same ligand [21].

In endothelial cells, soluble TGFβ1 interacts with type II receptor (TGFβ1RII) and, among the different types of ALKs, ALK-5 phosphorylation induces the activation of SMAD2/3 pathway involved in endothelial cells inhibition of proliferation/migration and in the promotion of the extracellular matrix proteins synthesis, while ALK-1 phosphorylation induces the activation of SMAD1 and SMAD5 pathways that leads to endothelial cells migration and proliferation [22].

ECPCs might contribute substantially to the overexpression of TGFβ that forces the maintenance of a dysregulated reparative process [23]. Thus, it has been demonstrated, in lung fibroblasts, that TGFβ1 induces overexpression of αvβ3 integrin which potentiates TGFβ1 responsiveness of the cells [24]. Although our findings are not completely exhaustive, indicate that ALK-5 expression is upregulated by exogenous TGFβ1 treatment, but that the downstream activation of signal transduction, through the ALK-5 receptor, may depend on αvβ3-mediated release of endogenous TGFβ1. Moreover, it is known that the type I receptor, in addition to Smad 2/3 protein phosphorylation, activates other non-Smad signaling pathways involving ERK1/2, TGF-β-activated kinase-1 (TAK-1), JNK, p38, Rho GTPases, and the PI3 K–AKT pathways [25]. We observed that while the interaction between the RGD antagonist and the integrin receptor did not induce any downstream signal transduction activation, the co-treatment with triazole RGD antagonist reduced significantly the ERK1/2 activation induced by TGFβ1.

TGFβ1, being the most important mediator in tissue fibrosis, is recognized to induce in vitro the endothelial–mesenchymal transition in endothelial cells and circulating endothelial precursor cells [26–29]. Despite other authors demonstrated that a cyclic RGD antagonist enhances in vitro and in vivo vascularization in a model of EPCs [30], we found a mild but significant reduction in the capillary formation after the treatment with the triazole RGD antagonist. This result was in agreement with our previous observation on mature human umbilical vein endothelial cells (HUVEC) [31]. We suggest that our findings depend by the contribution of two main different mechanisms of interaction between the RGD-2 antagonist and the αvβ3 receptor. The first is the direct role that integrin αvβ3 exerts in endothelial cells invasion and in vitro angiogenesis, resulting in a reduction in invasion and angiogenesis operated by the linear triazole peptidomimetic antagonists alone. The second is the interfering effect of RGD-2 antagonist in the TGFβ1/αvβ3 crosstalk that reverts EndoMT in ECPCs and exerts a fundamental anti-fibrotic and pro-resolution effect. It has to be noted that the interest in the synthesis and identification of bioactive RGD antagonists originates from the need in improving anti-angiogenic treatment of cancer, since the outcomes of drugs against vascular endothelial growth factor and relative receptors have shown a partial lack of efficacy both in vitro and in vivo [32–34]. The αv RGD mimetic cyclic pentapeptide, Cilengitide (EMD 121974), has been identified as the first synthesized anti-angiogenic small molecule, and its effect on endothelial cells has been demonstrated [35]. Cilengitide showed encouraging activity in patients with glioblastoma and melanoma, and is currently used in clinical trials [36, 37]. In contrast to this, other authors found that Cilengitide enhances angiogenesis, and promotes tumor growth and cell invasion [38]. In our model, despite the strong effect of the RGD triazole on the inhibition of cell adhesion, we found a weak effect on the inhibition of invasion and angiogenesis that might be explained by the specific and unique activity of this linear triazole RGD antagonist against the αvβ3 receptor. Along with this, we should take into account that invasiveness and tube formation assays are performed on Matrigel substrate, against which the RGD triazole effect is not specific, and that the spatial occupation of the integrin receptor binding site by the RGD triazole may not be sufficient to overcome integrin redundancy in invasiveness and tube formation processes. Indeed, during the ECPCs invasion and migration process, many interactions between ECM and other integrin receptors, as α5β1 and α6β1, have been found to be involved [39–41], as well as many interactions between ECM and cell-derived proteases. Thus, the αvβ3 integrin receptor activity might be only partially involved in ECPCs invasion and tube formation [42].

**Fig. 5:**
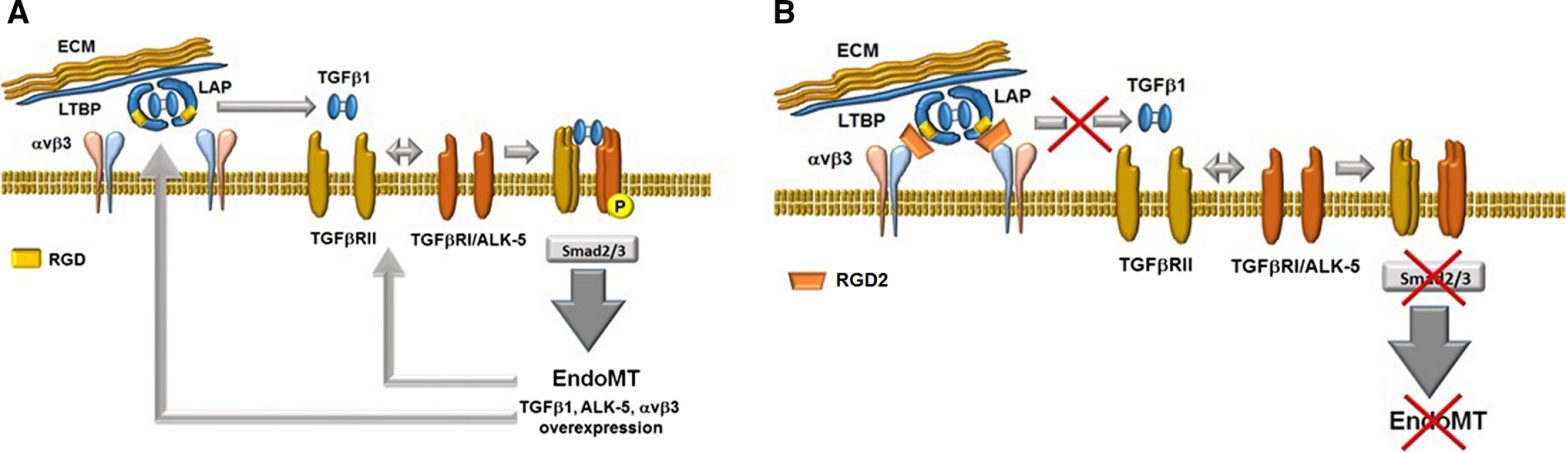
Hypothesis for molecular mechanism of RGD antagonist EndoMT reversion in ECPCs. **a** Mechanism of TGFβ1-induced EndoMT and αvβ3-mediated autocrine loop of TGFβ1 release in ECPCs. **b** Role of RGD-2 triazole antagonist in dampening autocrine loop of TGFβ1 activation

In a fibrotic lesion, TGFβ1 is released by activated fibroblasts and macrophages, or endothelial cells themselves, and induces a local overload of TGFβ1 driving to stroma activation. Circulating precursor endothelial cells, recruited by pro-inflammatory cytokines, differentiate in activated fibroblasts, and endogenous TGFβ1 amplifies the fibrotic vicious circle. To date, the therapeutic interventions on fibrotic diseases are the use of anti-inflammatory mediators, immunomodulatory drugs, or agents that directly block TGFβ1 activity, such as specific antibody. Unfortunately, anti-inflammatory treatment only retards but does not resolve fibrosis, while TGFβ1 antibody might compromise important activity of this cytokine in other tissues [43]. Our findings are in agreement with the importance of integrin involvement in fibrosis [44, 45]. Here, we introduced a new synthesized non-peptidic compound with anti-fibrotic properties in an in vitro model of tissue fibrosis. This RGD antagonist not only prevents the αvβ3-mediated TGFβ1 release dampening the autocrine loop of ECPCs activation, but also promotes a mesenchymal-to-endothelial transition (MEndoT), reverting the TGFβ1-mediated phenotype of activated endothelial cells. It should be noted that a αvβ6 monoclonal antibody is involved in a phase 2 study for idiopathic pulmonary fibrosis (STX-100, NCT01371035) [46]. This observation confirms the need of further investigations to delineate the role that integrin antagonists might have in the designing of anti-fibrotic therapies. On the whole, our results might support the hypothesis for an EndoMT reversion mediated by RGD antagonist. Exogenous TGFβ1, released in the fibrotic microenvironment, induces αvβ3 receptor overexpression in ECPCs. The presence of high levels of αvβ3 receptors enhances the release of active TGFβ1 from LAP. Endogenous ECPCs-derived TGFβ1 interacts with its receptors on ECPCs themselves promoting EndoMT (Fig. 5a). The RGD antagonist, by occupying the binding site of the αvβ3 receptor, reduces the traction exerted by the heterodimer on the RGD sequence of the LAP, thus reducing endogenous TGFb1 release and dampening the autocrine loop of TGFβ1 activation (Fig. 5b).

## Materials and methods

### Synthesis of triazole-based non-peptide RGD peptidomimetic

The triazole-based RGD ligand was achieved using the click chemistry by combining azide and alkyne in the Cu-catalyzed azide–alkyne cycloaddition, followed by acid-mediated hydrolysis of the protecting groups of side chain isosteres [16, 31, 47]. Three bioactive compounds were selected starting from a group of αvβ3 integrin ligands.

### Solid-phase integrin binding assay

The inhibition of ^125^I-echistatin-specific binding to αvβ3 integrins by RGD antagonists was evaluated as previously reported [47]. Briefly, ^125^I-echistatin with a specific activity of 2000 Ci/mmol was purchased from Perkin Elmer, and integrin αvβ3 from human placenta was purchased from Millipore. Purified αvβ3 integrin (Millipore) was diluted in coating buffer [20 mM Tris (pH 7.4), 150 mM NaCl, 2 mM CaCl2, 1 mM MgCl2, 1 mM MnCl2] at concentrations of 500 or 1000 ng/mL. Aliquots of αvβ3 integrin (100 lL/well) were added to a 96-well plate (Perkin Elmer), and plates were incubated overnight at 4 °C, followed by washings with blocking/binding buffer [20 mM Tris (pH 7.4), 150 mM NaCl, 2 mM CaCl_2_, 1 mM MgCl_2_, 1 mM MnCl_2_, and 1 % BSA], and then incubated at room temperature for an additional 2 h. After two washing with the same buffer, aliquots of ^125^I-echistatin (0.05 nM) were added to each well with different concentrations of RGD compounds (from 0.01 to 100 nM). Non-specific binding was defined as ^125^I-echistatin bound in the presence of an excess (1 μM) of unlabeled echistatin. After 3 h incubation at RT, plates were washed three times with blocking/binding buffer and counted in a Top-Count NXT microplate scintillation counter (Perkin Elmer) using 200 μL/well of MicroScint-40 liquid scintillation (Perkin Elmer). Data are shown as mean ± SD from three independent experiments. IC50 values were determined by fitting binding inhibition data by non-linear regression using GraphPad Prism 4.0 Software Package (GraphPad Prism, San Diego, CA).

### Isolation of endothelial colony-forming cells and culture conditions

Endothelial Colony-Forming Cells (ECPCs), a subpopulation of Endothelial Precursor Cells (EPCs), were isolated from >50 ml human umbilical cord blood (UCB) of health newborns, as described [48, 49], for the banking established by the Umbilical Cord Bank of Careggi Hospital (Florence, Italy) after maternal informed consent in accordance with the Declaration of Helsinki and in compliance with Italian legislation. ECPCs were analyzed for the expression of surface antigens (CD31, CD44, CD29, ULEX, KDR, and αvβ3) and were grown in complete EGM™-2 BulletKit™ (CC-3162 Lonza) with 10 % Fetal Bovine serum (FBS) (Hyclone). Confluent cell cultures were propagated every 3 days, for no more than 10 passage, and seeded on gelatin-coated tissue culture plates at a density of 5 × 10^5^ cells/cm^2^ in a 5 % CO2 humidified incubator at 37 °C. ECPCs cultures, grown to subconfluence, were treated for 24 h with 1 ng/mL of hrTGFβ1 (Peprotech), or triazole RGD antagonist (1 μM), or both in EGM-2 medium (Endothelial cells Growth Medium-2) in the absence of growth factors and in the presence of 2 % FBS (Fetal Bovine Serum). In some experiments, treated cells were exposed to fresh medium for an additional 24 h treatment before protein extraction.

### Flow cytometric analysis

Cells were collected using Accutase (a trypsin-free cell dissociation buffer from Sigma) and resuspended in PBS with 1 % BSA (FACS buffer). After blocking, cells were incubated with 1 μg/50 μL of primary antibody [anti-CD31, anti-CD44, or anti-CD29 (BD Biosciences PharMingen); anti-ULEX (Vector Laboratories), anti-KDR (RELIATech), or anti-αvβ3 (Millipore)] for 1 h at 4  °C, followed by 1 h incubation with FITC-conjugated secondary antibodies (Santa Cruz). After extensive washes using FACS buffer, cells were analyzed at 488 nm on the flow cytometer FACScan system (BDFACSCanto).

### Cell adhesion assay

Sub-confluent cultures of ECPCs between the 3rd and 6th passage were used for the inhibition of adhesion assay. Plates (96 wells) were coated with Matrigel™ Matrix (BD Biosciences) (10 μg/ml), vitronectin (10 μg/mL), fibronectin (1 μg/mL), or osteopontin (0.5 μg/mL), by overnight incubation at 4 °C. Plates were washed with PBS and then incubated at 37 °C for 1 h with PBS-1 % BSA. After being washed, ECPCs were counted, suspended in serum-free medium, and exposed to triazole RGD antagonists (final concentration was 0.01, 0.1, 1.0, or 10 μM) at 37 °C for 30 min to allow for the ligand-receptor equilibrium to be reached. ECPCs were plated ((4–5)10^4^ cells/well) and incubated at 37 °C for 2 h. All the wells were washed with PBS to remove the non-adherent cells, and 0.5 % crystal violet solution in 20 % methanol was added. After 2 h of incubation at 4 °C, plates were examined at 540 nm in an ELX800 counter (Bio TEK Instruments). Data were expressed as percentage of inhibition compared to untreated cells. Experiments were conducted in triplicate and were repeated at least three times [49].

### RNA extraction and RT-PCR

Total RNA was extracted from ECPCs using RNAgents (Total RNA Isolation System, Promega, Madison, WI) and quantified spectrophotometrically. A volume of 500 ng of RNA was then retrotranscribed using ImProm-II reverse transcriptase (Promega, Madison, WI). Aliquots of 2 μl of the cDNA were used for PCR amplification. The specific primers used for the identification of human αv, β3, and GAPDH were designed according to published human cDNA sequences in the Genbank database, using FastPCR software [50]: αv (forward5′-CTA TGA GCT GAG AAA CAA TGG TCC-3′ and reverse 5′GCT GCT CCC TTT CTT GTT CTT C-3′690-bp product); β3 (5′- GGG GAC TGCC TGT GTG ACT C-3′ and reverse 5′-CTT TTC GGT CGT GGA TGG TG-3′ 610-bp product); GAPDH (forward: 5′-ACC ACA GTC CAT GCC ATC AC-3′ and reverse: 5′-TCC ACC ACC CTG TTG CTG TA-3′, 452-bp product). PCR was carried out on a Perkin Elmer Thermal cycler. Ten microliters of each PCR products were visualized after electrophoresis in a 2 % agarose. cDNA products were evaluated on the basis of a standard PCR marker (Promega) and quantified by densitometric analysis using ImageJ software (NIH).

### Western blotting

ECPCs monolayers were lysed on ice in radioimmunoprecipitation assay buffer [50 mmol/L Tris–HCl (pH 7.5), 150 mmol/L NaCl, 1 % Triton X-100, 2 mmol/L EGTA, 1 mmol/L sodium orthovanadate, 1 mmol/L phenylmethane sulfonyl-fluoride, 10 μg/mL aprotinin, and 10 μg/mL leupeptin]. Samples were resolved by SDS-PAGE and transferred to a nitrocellulose membrane (Millipore, Billerica, MA). The blots were incubated in Bløk^®^-FL blocking buffer at room temperature and probed with primary antibodies and appropriate secondary antibodies for infrared fluorescence detection. Membranes were visualized by Odyssey Imager which quantifies protein amount proportional to infrared signal. Antibodies used for western blotting were as follows: anti-TGFβ1 (orb11468, Byorbit); anti-Alk5/TGFβRI (GTX102784, Genetex); anti-PhosphoERK1/2 and anti ERK1/2 (9101 and 9102, Cell Signalling); anti α-SMA (A2547, Sigma); anti-vimentin (V6630, Sigma); and anti-Phospho SMAD2 and anti-SMAD2/3 (3101 and 3102, Cell Signalling). Human anti-β-tubulin (Millipore 05-661) was used as a loading control. Fluorescent goat anti-mouse and anti-rabbit secondary antibody were conjugated to AlexaFluor-680 and AlexaFluor-750, respectively (Invitrogen). Fluorescent signals of protein bands were recorded at 700 nm (mouse) and 800 nm (rabbit); in some experiments, the quantification of immunoblotting bands was performed by densitometric analysis with Odyssey infrared imaging system (Li-COR Biosciences, USA).

### Immunofluorescence

ECPCs cells were cultured on 25-mm coverslips pre-coated with 10 μg/ml of VN. After 24 h of incubation, in the presence of TGFβ1, RGD-2 antagonist, or both, in complete EGM-2 medium, cells were washed in PBS, were fixed in 4 % paraformaldehyde, and membranes were permeabilized in 0.1 % Triton X-100 solution. Coverslips were incubated in blocking solution (PBS supplemented with 4 % BSA and 1 % horse serum) and then incubated at 4 °C overnight with anti-α-SMA primary antibodies, washed and incubated for 1 h with goat anti-mouse AlexaFluor-488 antibodies (Invitrogen). Cell nuclei were counterstained with DAPI (1 μg/ml for 10 min at 37 °C). Following two washes in PBS, coverslips were mounted with propylthiogallate on glass slides, and the cells were observed with an inverted confocal Nikon Eclipse TE2000 microscope equipped with a 960S-Fluor oil immersion lens.

### ECPCs migratory activity

In order to investigate the migratory activity of ECPCs, Boyden chamber assays were performed using Millicell cell culture inserts (Millipore 8-μm pore size, 12 mm diameter). A 3D barrier of 50 μg/cm^2^ of Matrigel was stratified on the filters, and ECPCs were loaded into the upper compartment (5 × 10^4^ cells/well) in 400 μl of complete EGM-2 containing TGFβ1, RGD-2 antagonist, or both and placed into 24-well culture dishes containing 600 μl of EGM-2 complete medium. After overnight incubation at 37 °C, non-invading cells were removed mechanically using cotton swabs, and micro-porous membrane containing the invaded cells was fixed in 96 % methanol and stained with Diff-Quick staining solutions. Migratory activity was evaluated by counting the cells which migrated toward the lower surface of the filters (six randomly chosen fields for each filter).

### In vitro tube formation assay

The effects of TGFβ1, RGD-2 antagonist, or both on the ability of ECPCs to reorganize and differentiate into capillary-like network were assessed, thereby Matrigel morphogenesis assay. Briefly, 50 μl of Matrigel (1 mg/ml) was added into wells of a 96-well plate and polymerized for 1 h at 37 °C. After 24 h of incubation, in the presence of TGFβ1, RGD-2 antagonist, or both, in complete EGM-2 medium, cells were washed once with PBS, harvested by trypsinization, and collected by centrifugation. Then, cells were resuspended in 200 μl of EGM-2 complete medium and placed into Matrigel-coated wells (6 × 10^5^ cells/well). After 12 h incubation on Matrigel at 37 °C, the plates were photographed under a phase contrast microscope. The degree of tubule formation was quantified by counting the branching points in four randomly chosen fields from each well [51].

### Wound healing assay

Cell migration was evaluated by an in vitro wound healing assay. Cells were grown at 80–90 % confluence in 35-mm dishes; the cell layer was wounded with a sterile 200-ml pipette tip and incubated in 0.1 % FBS culture medium for 24 h. The wound was observed after 18 h, and pictures were taken using phase contrast microscope. The degree of healing was quantified by measuring the distance between opposing edges of the wound. Four wound/treatment and three measurements/wound were taken. Percentage of inhibition was expressed compared to untreated cells.

### Statistical analysis

Results were analyzed using a 2-tailed Student’s *t* test to assess statistical significance. Statistical differences are presented at probability levels of *P* < 0.05 or *P* < 0.01.

## Abbreviations

ALK: Activin receptor‐Like Kinase
α-SMA: Alpha-Smooth Muscle Actin
αvβ3: Alphavbeta3 integrin
ECFC: Endothelial colony-forming cells
ECM: Extracellular matrix
EndoMT: Endothelial-to-mesenchymal transition
ERK1/2: Extracellular signal-regulated kinases 1/2
FN: Fibronectin
MEndoT: Mesenchymal-to-endothelial transition
RGD: Arginine-glycine-aspartic acid
OPN: Osteopontin
Smad: Small mothers against decapentaplegic homolog
TGF-β: Transforming growth factor-β
VN: Vitronectin
